# Evaluating the Probability of Head Acceleration Events in Elite Men’s and Women’s Rugby Union Match-Play: The Impact of Tackle Height and Body Position

**DOI:** 10.1007/s40279-025-02241-2

**Published:** 2025-05-07

**Authors:** Cameron Owen, Greg Roe, James Tooby, Thomas Sawczuk, James Brown, Matt Cross, Éanna Falvey, Sharief Hendricks, Simon Kemp, Lindsay Starling, Keith Stokes, Ross Tucker, Ben Jones

**Affiliations:** 1 Carnegie Applied Rugby Research (CARR) Centre, Carnegie School of Sport, Leeds Beckett University, Leeds, UK; 2 England Performance Unit, Rugby Football League, Manchester, UK; 3 Obesity Institute, Leeds Beckett University, Leeds, UK; 4 Division of Physiological Sciences, Department of Human Biology, Faculty of Health Sciences, University of Cape Town, Cape Town, South Africa; 5 The Division of Sport and Exercise Medicine (DiSEM), Department of Exercise, Sport and Lifestyle Medicine, Faculty of Medicine and Health Sciences, Stellenbosch University, Stellenbosch, South Africa; 6 Premiership Rugby, London, UK; 7 World Rugby, Dublin, Ireland; 8 School of Medicine and Health, University College Cork, Cork, Ireland; 9 Rugby Football Union, Twickenham, UK; 10 London School of Hygiene and Tropical Medicine, London, UK; 11 Centre for Health and Injury and Illness Prevention in Sport, University of Bath, Bath, UK; 12 UK Collaborating Centre on Injury and Illness Prevention in Sport (UKCCIIS), University of Bath, Bath, UK; 13 Faculty of Health Sciences, School of Behavioural and Health Sciences, Australian Catholic University, Brisbane, QLD Australia

## Abstract

**Background:**

Head acceleration events (HAEs) are an increasing concern in collision sports owing to potential negative health outcomes.

**Objectives:**

The objective of this study is to describe the probabilities of HAEs in tackles of differing heights and body positions in elite men’s and women’s rugby union.

**Methods:**

Instrumented mouthguards (iMGs) were worn in men’s (*n* = 24 teams, 508 players, 782 observations) and women’s (*n* = 26 teams, 350 players, 1080 observations) rugby union matches. Tackle height (i.e. point of contact on ball-carrier) and body positions of tacklers and ball-carriers were labelled for all tackles in which a player wore an iMG. HAEs from the initial impact were identified. Mean player, tackler and ball-carrier exceedance probabilities for various peak linear and angular acceleration thresholds were estimated from ordinal mixed-effects models.

**Results:**

Contact with ball-carriers’ head/neck resulted in the highest mean HAE probabilities for both sexes. The probability of an HAE to the ball-carrier decreased as tackle height lowered. The highest probability for the tackler was initial contact to the ball-carriers upper leg. Body position influenced the probability of HAEs, with falling/diving ball-carriers resulting in higher mean probabilities. When a player, regardless of role, was bent-at-waist, elevated HAE probabilities were observed in men’s competitions. Women’s data demonstrated similar probabilities of an HAE for all body positions.

**Conclusions:**

Initial contact to the ball-carrier’s head/neck had the highest chance of an HAE, whilst role-specific differences are apparent for different tackle heights and body positions. Future player-welfare strategies targeting contact events should therefore consider HAE mechanisms along with current literature.

**Supplementary Information:**

The online version contains supplementary material available at 10.1007/s40279-025-02241-2.

## Key Points

**Table:** 

Understanding which mechanisms are associated with head-acceleration events is important when considering how to mitigate them.
The greatest risk of a head acceleration event is when an initial impact is made to the ball-carriers head/neck; however, the ball-carrier and tackler are observed to have different risk profiles by tackle height, and, therefore, the focus of interventions should be on avoiding initial contact to the ball-carriers head/neck without promoting tackling the lower body.
Men’s players are at the greatest risk in the tackle when they are bent at the waist, whilst the probabilities of an HAE are similar across body positions in women’s competitions; therefore, both the ball-carrier and tackler should aim to have more upright postures whilst avoiding initial contact to the ball-carriers head/neck to minimise head-acceleration events.

## Introduction

Head acceleration events (HAEs) are a resultant acceleration of the head in response to direct or indirect contact [1] and are therefore a frequent occurrence in contact sports such as rugby union [2]. It has been posited that the brain can experience significant loading during contact events, even in the absence of a concussion, as the rapid acceleration and subsequent deceleration of the skull results in deformation of brain matter and possible traumatic brain injury [3, 4]. As such, HAEs may have immediate implications for clinical presentation at the time of head acceleration (i.e. concussion with clinical signs/symptoms), medium-term implications since the accumulation of head accelerations may be associated with clinical outcomes (i.e. greater frequency of HAEs on the day is associated with a concussion in American Football) [5] and long-term implications as a result of proposed associations with later-in-life neurodegenerative outcomes [6–8]. The recent adoption of mouthguards containing accelerometers and gyroscopes (i.e. instrumented mouthguards [iMG]) globally within several collision sports has provided a valid, non-invasive method for quantifying the HAEs of players in vivo [2, 9, 10]. Therefore, to support the development of athlete welfare initiatives in preventing short- to medium-term clinical outcomes and supporting the long-term brain health of players, a detailed understanding of event characteristics associated with HAEs is required to help inform interventions such as law or tackle technique changes [11].

Rugby union involves intermittent high-intensity activities including contact events (e.g. rucks, tackles and ball-carries) [12–14]. The highest proportion of injuries occur during contact events, particularly the tackle [15–19]. In recent years, player welfare-driven law changes have focused on reducing the number of head injuries that occur when tackling or being tackled (e.g. as the ball-carrier) [15–18, 20, 21]. Law changes include attempts to lower the maximum height of a legal tackle and increasing the sanction for head contact at the existing legal tackle height [22]. These interventions were based on video analysis studies that showed that upright tacklers contacting upright ball-carriers, and contact above the sternum of the ball-carrier, increased the risk of a head injury to both players [23, 24].

The greatest risk of head injury is observed during tackles that contact the ball-carrier high [19, 20]. In addition, there is also a head injury risk to the tackler, although to a lesser extent, when they contact the ball-carrier below the hips, on the ball-carrier’s upper legs and knees [21, 22]. Therefore, an optimal tackle height that places the tackler’s head neither too high nor too low, but in contact with or in proximity to the ball-carrier’s torso may reduce the risk of head injury. Since tackle height has been suggested to be modifiable [25], strategies have been adopted to lower the maximum height of the legal tackle to achieve an overall reduction in risk. It is not known, however, how tackle height influences the probability of experiencing an HAE and/or the magnitude of an HAE dependent on player role in the tackle.

Comprehensive assessments of the overall exposure of HAEs in rugby union have been performed [2, 26], but less is known about HAEs experienced as a consequence of tackles with specific characteristics [27]. The incidence of HAEs for men’s and women’s have been reported (e.g. forwards, ≥ 25 g; 3.92/1000 h [95% confidence intervals: 3.34–4.51] and 1.57/1000 h [1.16–1.99], and backs, ≥ 25 g; 3.20/1000 h [2.20–4.19] and 0.71/1000 h [0.43–0.99]), although such rates include HAEs from all contact events, e.g. tackles, rucks and mauls [2]. When considering the tackle specifically, for men, the probability of at least one HAE being recorded is 76.7% (74.5, 78.7), and the probability of exceeding a peak linear acceleration (PLA) of 25 g is 18.9% (17.2–20.9) and of 55 g is 1.4% (1.1–1.8) [26]. Similar trends are also observed for peak angular acceleration (PAA) [26]. The overall probability of an HAE from a tackle within women’s competitions are yet to be reported. It is important to quantify the probability of specific mechanisms, in addition to the incidence of HAEs, to identify which aspects of the game have the greatest risk and to assess if subsequent interventions change the risk of an injury occurring.

To date, the only study assessing specific tackle characteristics and HAEs in rugby union suggests impact to the ball-carrier above the sternum results in the greatest chance of an HAE occurring to the ball-carrier (HAE > 30 g for men and women, above sternum 8.7% and 5.6%, torso 0.3% and 0.1% and legs 1.1% and 1.6%) [27]. By contrast, it is greatest for the tackler when contact is made with the ball-carrier legs (HAE > 30 g for men and women, above sternum 4.1% and 2.8%, torso 4.0% and 1.5% and legs 10.1% and 8.0%) [27]. However, tackle height was categorised as ‘low’ (legs), ‘medium’ (torso) or ‘high’ (above sternum), which is less granular than may be optimal when considering potential tackle height law/rule changes. The study also did not consider the body position of the players in the tackle. Finally, the hierarchy of the data structure was not considered [27], which is important, as failure to account for lack of independence of observations can result in biased estimates and increase the risk of Type 1 errors [28]. Further investigation of the occurrence of HAEs during tackle events whilst addressing such limitations could be beneficial to inform future player-welfare interventions. Therefore, the aim of this study was to describe the probability of a player experiencing an HAE during the initial collision for different tackle heights and body positions and to consider differences between players’ roles in the tackles using a global dataset for elite men’s and women’s rugby union players.

## Methods

### Study Design and Participants

A prospective observational cohort study was conducted in professional men’s (Currie Cup [*n* = 8 teams, 141 players, 341 observations], Premiership Rugby [*n* = 11 teams, 131 players, 306 observations] and Super Rugby [*n* = 5 teams, 63 players, 135 observations]) and semi-professional women’s (Premiership Women’s Rugby [*n* = 10 teams, 104 players, 428 observations] and Farah Palmer Cup [*n* = 13, 246 players, 652 observations]) rugby union players competing during the 2023 season. Institutional ethics approval was received, and player informed consent obtained (ref. no.: 108638).

### Instrumented Mouthguards

All players used custom-fit iMGs (V1.4, Prevent Biometrics, Minneapolis, MN, USA) as previously outlined [26]. Laboratory validation of this device yielded a concordance correlation coefficient of 0.98 (95% confidence interval [CI]: 0.9–0.99). Following the filtering of accelerometer and gyroscope signals of each HAE using a four-pole, zero phase, low-pass Butterworth filter with a cut-off frequency (− 6 dB) of 200 Hz, an in-house Prevent Biometrics machine learning algorithm determined the level of noise remaining in each HAE signal as containing minimal (*n* = 5121), moderate (*n* = 272) or severe (*n* = 95) noise. HAEs classified with moderate and severe noise were filtered with an additional filter with cut-off frequencies of 100 and 50 Hz, respectively. Linear kinematics were transformed to the head centre of gravity using the relative acceleration equation [29]. Following post-processing by Prevent Biometrics, PLA and PAA were extracted from the timeseries data of each HAE.

### Video Analysis

Opta data were provided by StatsPerform (Chicago, IL) for all matches of the 2023 season across each competition. These data included video timestamps, player identifiers and player role (i.e. ball-carrier or tackler) for each player’s involvement within a tackle event. Player events occurring in the same tackle event were grouped together via a common contact event ID. Additional video analysis was conducted to add detail to each coded tackle event, including the initial point of contact on the ball-carrier (i.e. tackle height; head/neck, shoulders, torso, upper leg [hip to above knee] and lower leg [knee to foot]) and the body position of the ball-carrier and tackler (upright [the player has no visible bend at the waist], bent at waist [the player has made an attempt to drop their tackle height by bending at the hips] and falling/diving [players feet have left the ground and they are no longer supporting their body weight]). Both labels described the tackle characteristics at the initial point of contact of the specific tackler being labelled. For example, if a ball-carrier was tackled by three players, initial point of contact and body position would be labelled three times, describing the point of initial contact and body position at the initial contact by each respective tackler. This analysis was conducted by 13 analysts (Currie Cup, *n* = 5; Premiership Women’s Rugby and Premiership Rugby, *n* = 1; Farah Palmer Cup, *n* = 1 and Super Rugby, *n* = 6). Inter-rater reliability, assessed through Krippendorf’s alpha [30], was 0.77 (95% confidence interval; 0.72, 0.81) for tackle height, 0.52 (0.45, 0.60) for tackler body position and 0.68 (0.64, 0.73) for ball-carrier body position. Intra-rater reliability, assed by Cohen’s kappa [31], ranged from 0.57–0.89 for tackle height, 0.49–0.86 for tackler body position and 0.51–0.93 for ball-carrier body position.

To link HAEs to the tackle events which caused them, all HAEs were synchronised to video footage using a custom-built MATLAB graphical user interface, enabling a synchronisation to within a tolerance of two frames to locate the causing event of each HAE within the video. Subsequently, each HAE was labelled to identify the instrumented player event (i.e. the event the player was completing when experiencing the HAE; e.g. ball-carry, tackle, ruck or maul) and the causing event (i.e. the inflicting event of opposition player; initial, secondary, grounding or ruck [32]). For example, if an HAE was recorded for a player carrying the ball, the instrumented player event was labelled as the coded ball-carry event, and the causing event was labelled as the tackle event which triggered the HAE and when it occurred in the tackle.

Tackle involvements which corresponded with an ‘on-the-teeth’ proximity signal (i.e. the proximity threshold for the device is met and indicates the player is wearing it) were considered for analysis (*n* = 19,472). Only HAEs triggered by the initial collision with an opposition player in the tackle event were used in data analysis (*n* = 5488). These HAEs were used because they could be linked directly to the labelled tackle characteristics of a specific tackler rather than allowing for any ambiguity for who has caused the HAE (i.e. in a two-person tackle, initial contact to the ball-carriers legs by the first tackler did or did not result in an HAE, the second tackler who made contact at the shoulder did or did not result in an HAE). Initial head impacts are also the most likely focus of any interventions regarding tackle height and player body positions (i.e. initial contact to the head is penalised as a high tackle). HAEs triggered during secondary contact, ground collision or breakdown stages of coded tackle events were not used in the analysis. The maximum PLA and PAA value from HAEs recorded for each initial collision between a ball-carrier and a tackler were used to describe head acceleration. If no HAE was recorded during the initial collision, the collision was denoted as ‘not recorded’.

### Data Analysis

All analyses were conducted in R (version 4.3.0) using the ordinal [33] and emmeans [34] packages. To estimate the probability of different tackle heights resulting in different ranges of HAE magnitudes, an ordinal mixed-effects regression model was used [35]. Exceedance probabilities (i.e. the probability that HAE magnitudes greater than a certain value would be experienced) were estimated at six ranges for PLA (recorded, ≥ 10 g, ≥ 25 g, ≥ 40 g, ≥ 55 g and ≥ 70 g) and PAA (recorded, ≥ 1000 rad/s^2^, ≥ 2000 rad/s^2^, ≥ 3000 rad/s^2^, ≥ 4000 rad/s^2^ and ≥ 5000 rad/s^2^) to align with previous literature in rugby union [26]. Exceedance probabilities for recorded, ≥ 25 g, ≥ 55 g, ≥ 2000 rad/s^2^ and ≥ 4000 rad/s^2^ are presented in the results section. The remaining exceedance probability thresholds (i.e. ≥ 10 g*,* ≥ 40 g*,* ≥ 70 g*,* ≥ 1000 rad/s^2^, ≥ 3000 rad/s^2^ and ≥ 5000 rad/s^2^) for both tackle height (Supplementary 1 and 2) and body position (Supplementary 3, 4, 5 and 6) are provided in the same format within the supplementary material for the reader to use as reference data owing to the trends being similar.

For the tackle height model, initial impact on the ball-carrier was interacted with player role and sex. Fixed effects predicted the probability of each HAE magnitude range occurring within a single collision event for each sex and role combination. In each model, player ID was nested within match ID and included as a random effect to account for repeated measurements within players and within matches. Contact-event ID was also included as a random effect to account for the cross-classification of player events within the same contact event [36, 37].

For the purposes of modelling player body positions, ball-carrier and tackler body position (BC-T) were grouped to create nine interactions. Separate models were run for men’s and women’s body position owing to few observations in the women’s falling/diving-upright tackle types (*n* = 5) resulting in Hessian singularity. Therefore, falling/diving-upright was removed as a factor from the women’s model. Models were run for each sex, with the ball-carrier–tackler body position interaction treated as a fixed effect that interacted with role. The random effect structure was the same as outlined for the tackle-height models.

Role-specific estimated marginal means were extracted from the models conditional on player role and the tackle height/body position interaction. Pooled mean probabilities for a player to receive an HAE were extracted conditional on the tackle height/body position alone, without distinguishing for player role. Median exceedance probabilities and 95% confidence intervals (CIs) for all estimates were produced via a bootstrapping approach with 1000 resamples applied to the modelling process. Differences were indicative when the confidence intervals did not overlap [38], as pairwise comparisons of ordinal regression models could not be determined.

## Results

Overall, 18,087 tackles (men; *n* = 7429, women; *n* = 10,658) were included in the analysis. There were 8956 tackles effected on a ball-carrier who was wearing an iMG (men; *n* = 3848, women; *n* = 5108) and 9131 tackles made by a defender who was wearing an iMG (men; *n* = 3581, women; *n* = 5550). In total, 3465 initial HAEs were recorded (men; *n* = 2064, women; *n* = 1401). A total of 245 tackles with an associated HAE were removed owing to inconclusive tackle-height or body-position labels due to the camera angle. Table 1 presents the number and proportion of tackles and initial HAEs recorded by tackle height and body position interactions.

**Table 1:** Number and proportion of tackles and initial head-acceleration events for men’s and women’s rugby union

	Men		Women	
	Tackles (*n* [% of total])	HAEs (*n* [% of total])	Tackles (*n* [% of total])	HAEs (*n* [% of total])
Tackle height				
Head/neck	150 (2.01%)	58 (2.81%)	259 (1.64%)	67 (4.78%)
Shoulder	2679 (36.06%)	834 (40.41%)	2862 (26.85%)	459 (32.76%)
Torso	3170 (42.67%)	806 (39.05%)	5127 (48.10%)	565 (40.33%)
Upper leg	1308 (17.61%)	347 (16.81%)	2181 (20.46%)	282 (20.13%)
Lower leg	122 (1.64%)	19 (0.92%)	229 (2.15%)	28 (2.00%)
Body position interaction (ball-carrier–tackler)				
Upright–upright	630 (8.48%)	158 (7.66%)	747 (7.01%)	83 (5.92%)
Upright–bent-at-waist	858 (11.60%)	245 (11.9%)	1092 (10.20%)	105 (7.49%)
Upright–falling/diving	394 (5.30%)	83 (4.02%)	1011 (9.49%)	138 (9.85%)
Bent-at-waist–upright	487 (6.56%)	141 (6.83%)	484 (4.54%)	60 (4.28%)
Bent-at-waist–bent-at-waist	4107 (55.30%)	1233 (59.70%)	4929 (46.20%)	652 (46.50%)
Bent-at-waist–falling/diving	556 (7.61%)	124 (6.01%)	2175 (20.4%)	344 (24.6%)
Falling/diving–upright	36 (0.48%)	12 (0.58%)	5 (0.05%)	0 (0.00%)
Falling/diving–bent-at-waist	240 (3.23%)	47 (2.28%)	93 (0.87%)	6 (0.43%)
Falling/diving–falling/diving	112 (1.51%)	21 (1.02%)	122 (1.14%)	13 (0.93%)

### Tackle Height

**Fig. 1:**
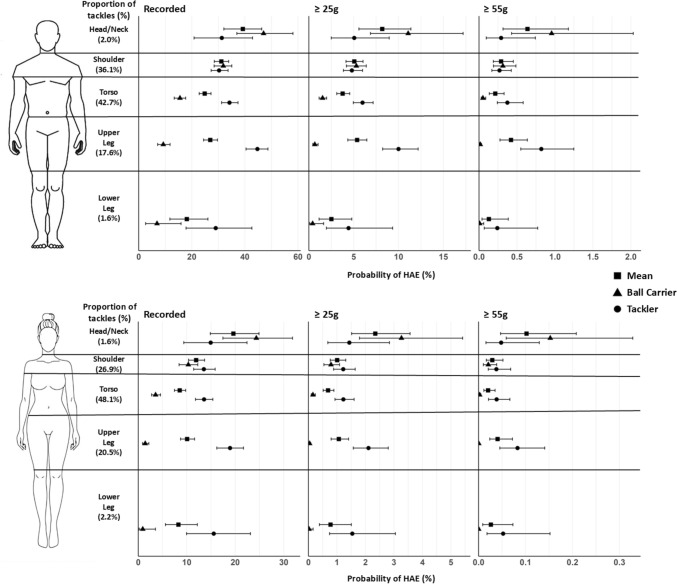
Peak linear acceleration exceedance probabilities for men’s (recorded, ≥ 25 g and ≥ 55 g) and women’s (recorded, ≥ 25 g and ≥ 55 g) rugby union players by tackle height for all tackle, ball-carrier and tackler head-acceleration events. Shape position indicates the median exceedance probability, with 95% confidence intervals shown by the error bars. Note all *x*-axes are different

**Fig. 2:**
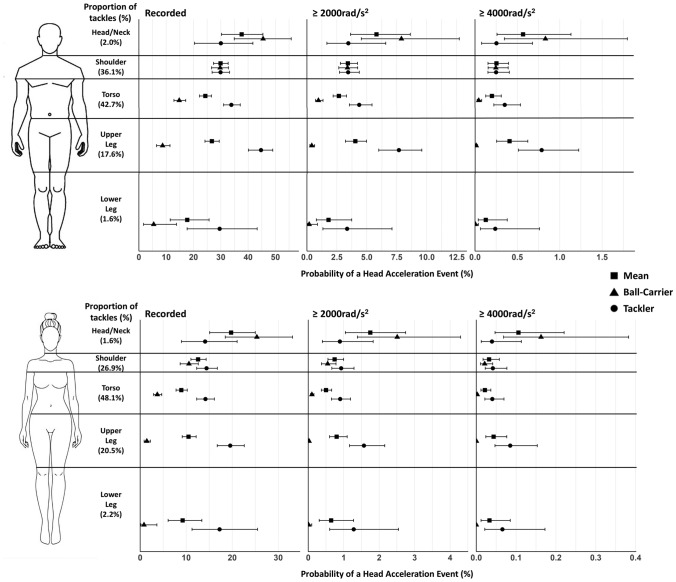
Peak angular acceleration exceedance probabilities for men’s (recorded, ≥ 2000 rad/s^2^ and ≥ 4000 rad/s^2^) and women’s (recorded, ≥ 2000 rad/s^2^ and ≥ 4000 rad/s^2^) rugby union players by tackle height for all tackle, ball-carrier and tackler head-acceleration events. Shape position indicates the median exceedance probability, with 95% confidence intervals shown by the error bars. Note all *x*-axes are different

Men’s and women’s exceedance probabilities for different tackle heights are shown for PLA and PAA thresholds in Figs. 1 and 2, respectively.

#### Mean Probability as a Function of Contact Point on the Ball-Carrier’s Body

In both men and women, the greatest mean exceedance probability for an HAE during a tackle occurred when initial contact was to the ball-carrier’s head/neck. As shown in Figs. 1 and 2, head-/neck-height tackles produce the highest ball-carrier HAE probability and comparable tackler HAE probability to other body regions, resulting in a greater mean HAE probability compared with lower tackles. This was true across all magnitude thresholds for both PLA and PAA.

For men, the probability of an HAE for head/neck contact in the tackle was greater compared with the torso, upper leg and lower leg for recorded HAEs (Fig. 1; 39.36% versus 18.33–27.02%). At ≥ 25 g in men, differences were observed only between head/neck (8.15% [5.52, 11.58]) and the torso (3.76% [3.12, 4.52]) and lower leg (2.42% [1.09,4.93]), and at ≥ 55 g, no differences were observed. For women, HAE probability from contact to head/neck was greater than from initial impact to all other areas of the body for recorded and ≥ 25 g (Fig. 1; e.g. for ≥ 25 g*,* 2.35% contact to head/neck versus 5.21–7.36%). However, at higher magnitudes, only the shoulder (≥ 55 g; 0.03% [0.02, 0.05]) and torso (≥ 55 g; 0.02% [0.01, 0.04]) were lower than the head/neck (≥ 55 g; 0.10% [0.05, 0.22]).

#### Probability of a Ball-Carrier HAE as a Function of Tackle Height

For both men and women, the ball-carrier had the greatest exceedance probabilities when initial impact was made to the head/neck, with lower exceedance probabilities as initial contact was made further down the body across all PLA and PAA magnitudes (Fig. 1; ≥ 25 g women, 3.19% versus 0.02–0.79% and men, 11.04% versus 0.42–5.28%; Fig. 2; ≥ 2000 rad/s^2^ women, 2.52% versus 0.01–0.55% and men, 7.86% versus 0.18–3.38%). However, no differences were observed between the head/neck and shoulder ≥ 55 g (0.95% [0.42, 2.01] versus 0.31% [0.19, 0.49]) and ≥ 4000 rad/s^2^ (0.82 [0.35, 1.77] versus 0.25% [0.15, 0.39]) for men (Figs. 1, 2).

For recorded, ≥ 25 g and ≥ 2000 rad/s^2^ in men’s and women’s competitions, greater probabilities were also observed for shoulder compared with torso impact (e.g. Fig. 1; ≥ 25 g women, 0.79% [0.55, 1.09] versus 0.16% [0.10, 0.24] and men, 5.23% [4.19, 6.41] versus 1.51% [1.13, 1.99]) and for torso compared with the upper leg impact (e.g. Fig. 1; ≥ 25 g women, 0.16% [0.10, 0.24] versus 0.04% [0.02, 0.08] and men, 1.51% [1.13, 1.99] versus 0.64% [0.40, 0.10]).

#### Probability of a Tackler HAE as a Function of Tackle Height

The highest HAE probabilities for the tackler were observed when initial contact was made to the upper leg of the ball-carrier for both men (e.g. ≥ 25 g; 10.06% [8.12, 2.15]) and women (e.g. ≥ 25 g; 2.09% [1.57, 2.76]). Tackler HAE probability following contact to the upper leg was meaningfully higher than following shoulder contact across all HAE magnitudes, and following torso contact for all magnitudes except ≥ 55 g and ≥ 4000 rad/s^2^ for men’s tacklers. For a women’s tackler, the recorded exceedance probabilities were greater for the upper leg (e.g. PLA; 18.07% [16.18, 21.72]) when compared with the shoulder (e.g. PLA; 13.58% [11.49, 15.78]) and torso (e.g. PLA; 13.52% [11.81, 15.41]), but no other differences were observed between other tackle heights and exceedance thresholds (Figs. 1, 2).

### Body Position

The exceedance probabilities for the PLA and PAA by body position are presented in Tables 2 and 3 for men and in Tables 4 and 5 for women.

**Table 2:** Peak linear acceleration exceedance probabilities (recorded, ≥ 25 g and ≥ 55 g) for men’s rugby union players by ball-carrier–tackler body position interaction for all tackle, ball-carrier and tackler head-acceleration events

			Tackler body position								
			Mean probability			Ball-carrier probability			Tackler probability		
			Upright	Bent at waist	Falling/diving	Upright	Bent at waist	Falling/diving	Upright	Bent at waist	Falling/diving
Ball-carrier body position	Rec.	Upright	22.87% (19.23, 26.59)	26.45% (23.28, 29.81)	17.67% (14.33, 21.42)	20.66% (16.56, 25.62)	15.02% (11.8, 18.74)	6.13% (3.51, 9.86)	25.07% (20.45, 30.60)	38.14% (32.67, 43.16)	29.27% (23.37, 36.11)
		Bent at waist	26.66% (22.84, 30.76)	29.91% (28.05, 31.78)	23.1% (19.74, 26.71)	27.70% (22.5, 33.93)	23.35% (21.18, 25.64)	10.61% (7.49, 14.96)	25.78% (20.52, 31.48)	36.61% (34.07, 39.17)	35.67% (30.33, 41.86)
		Falling/diving	38.61% (24.10, 52.06)	19.19% (14.45, 24.64)	18.25% (11.94, 25.87)	52.77% (29.61, 73.61)	19.59% (13.10, 27.61)	13.10% (6.24, 23.66)	24.23% (9.86, 45.54)	18.50% (12.62, 26.21)	23.29% (14.01, 34.95)
	≥ 25 g	Upright	3.61% (2.65, 4.69)	5.17% (4.12, 6.46)	2.94% (2.05, 4.10)	3.00% (2.06, 4.35)	1.79% (1.21, 2.57)	0.46% (0.2, 0.98)	4.16% (2.89, 5.92)	8.59% (6.52, 10.87)	5.36% (3.61, 7.82)
		Bent at waist	4.63% (3.48, 5.94)	5.84% (5.08, 6.64)	4.34% (3.27, 5.59)	4.89% (3.35, 7.16)	3.68% (3.07, 4.46)	1.04% (0.60, 1.83)	4.34% (2.91, 6.15)	7.99% (6.89, 9.21)	7.72% (5.66, 10.1)
		Falling/diving	10.28% (4.24, 18.95)	2.69% (1.71, 4.11)	2.63% (1.35, 4.83)	16.10% (5.51, 33.08)	2.76% (1.46, 4.97)	1.45% (0.49, 3.82)	3.97% (0.95, 12.18)	2.52% (1.36, 4.58)	3.64% (1.60, 7.52)
	≥ 55 g	Upright	0.19% (0.10, 0.33)	0.38% (0.23, 0.6)	0.18% (0.09, 0.33)	0.15% (0.08, 0.27)	0.07% (0.04, 0.13)	0.01% (0.00, 0.03)	0.23% (0.13, 0.45)	0.69% (0.41, 1.12)	0.34% (0.17, 0.64)
		Bent at waist	0.28% (0.16, 0.47)	0.41% (0.27, 0.58)	0.30% (0.17, 0.53)	0.30% (0.16, 0.56)	0.20% (0.12, 0.30)	0.03% (0.01, 0.08)	0.25% (0.12, 0.46)	0.62% (0.42, 0.89)	0.58% (0.33, 0.99)
		Falling/diving	1.07% (0.25, 3.38)	0.13% (0.06, 0.26)	0.13% (0.04, 0.34)	1.85% (0.33, 6.68)	0.13% (0.05, 0.33)	0.05% (0.01, 0.21)	0.21% (0.03, 1.25)	0.11% (0.04, 0.26)	0.19% (0.06, 0.60)

Median exceedance probabilities and 95% confidence intervals are reported

*Rec.* recorded

**Table 3:** Peak angular acceleration exceedance probabilities (recorded, ≥ 2000 rad/s^2^ and ≥ 4000 rad/s^2^) for men’s rugby union players by ball-carrier–tackler body position interaction for all tackle, ball-carrier and tackler head-acceleration events

			Tackler body position								
			Mean probability			Ball-carrier probability			Tackler probability		
			Upright	Bent at waist	Falling/diving	Upright	Bent at waist	Falling/diving	Upright	Bent at waist	Falling/diving
Ball-carrier body position	Rec.	Upright	23.30% (19.98, 27.12)	27.82% (24.74, 30.98)	17.56% (13.92, 21.32)	20.66% (16.58, 25.48)	15.77% (12.71, 19.44)	5.54% (2.87, 9.77)	26.11% (21.53, 31.04)	39.65% (34.71, 44.73)	29.58% (23.5, 36.52)
		Bent at waist	26.73% (23.00, 31.05)	29.68% (27.85, 31.87)	22.45% (19.12, 26.08)	26.67% (21.32, 33.01)	22.28% (20.01, 24.54)	10.33% (7.23, 14.43)	26.81% (21.69, 33.03)	37.30% (34.68, 39.71)	34.56% (29.17, 40.59)
		Falling/diving	34.05% (19.81, 47.80)	19.62% (14.52, 25.49)	17.99% (11.86, 25.61)	45.32% (24.37, 66.73)	19.91% (13.48, 27.88)	12.8% (5.98, 23.74)	22.03% (8.32, 40.71)	18.83% (12.67, 26.48)	22.94% (13.40, 35.75)
	≥ 2000 rad/s^2^	Upright	2.16% (1.55, 2.97)	3.49% (2.65, 4.50)	1.75% (1.07, 2.65)	1.71% (1.10, 2.62)	1.07% (0.68, 1.61)	0.03% (0.01, 0.09)	2.64% (1.73, 3.83)	5.91% (4.47, 7.77)	3.29% (2.12, 5.18)
		Bent at waist	2.75% (1.97, 3.76)	3.59% (2.96, 4.31)	2.49% (1.75, 3.53)	2.72% (1.77, 4.17)	1.95% (1.50, 2.50)	0.52% (0.28, 0.93)	2.74% (1.82, 4.23)	5.18% (4.29, 6.27)	4.49% (3.12, 6.22)
		Falling/diving	5.11% (1.71, 11.41)	1.57% (0.87, 2.61)	1.44% (0.69, 2.75)	7.80% (2.38, 19.64)	1.60% (0.78, 2.99)	0.75% (0.21, 2.27)	1.88% (0.36, 6.40)	1.45% (0.68, 2.73)	2.05% (0.79, 4.90)
	≥ 4000 rad/s^2^	Upright	0.13% (0.06, 0.25)	0.28% (0.15, 0.50)	0.12% (0.05, 0.24)	0.09% (0.04, 0.19)	0.05% (0.02, 0.10)	0.00% (0.00, 0.02)	0.17% (0.08, 0.33)	0.52% (0.29, 0.94)	0.23% (0.10, 0.48)
		Bent at Waist	0.17% (0.09, 0.33)	0.27% (0.16, 0.45)	0.18% (0.09, 0.36)	0.17% (0.08, 0.36)	0.11% (0.06, 0.19)	0.02% (0.01, 0.05)	0.17% (0.08, 0.36)	0.42% (0.26, 0.70)	0.35% (0.18, 0.65)
		Falling/Diving	0.47% (0.09, 1.78)	0.08% (0.03, 0.19)	0.08% (0.02, 0.22)	0.78% (0.13, 3.28)	0.08% (0.03, 0.21)	0.03% (0.00, 0.14)	0.10% (0.01, 0.61)	0.07% (0.02, 0.20)	0.12% (0.03, 0.42)

Median exceedance probabilities and 95% confidence intervals are reported

*Rec.* recorded

**Table 4:** Peak linear acceleration exceedance probabilities (recorded, ≥ 25 g and ≥ 55 g) for women’s rugby union players by ball-carrier–tackler body position interaction for all tackle, ball-carrier and tackler head-acceleration events

			Tackler body position								
			Mean probability			Ball-carrier probability			Tackler probability		
			Upright	Bent at waist	Falling/diving	Upright	Bent at waist	Falling/diving	Upright	Bent at waist	Falling/diving
Ball-carrier body position	Rec.	Upright	9.26% (6.94, 12.41)	8.22% (6.33, 10.67)	8.64% (6.93, 10.94)	8.15% (5.5, 12.09)	3.01% (1.76, 4.85)	1.47% (0.76, 2.66)	10.32% (7.08, 14.35)	13.49% (10.1, 17.50)	15.81% (12.6, 19.90)
		Bent at waist	9.83% (7.17, 13.29)	10.27% (8.66, 12.15)	9.54% (7.81, 11.66)	10.35% (6.83, 14.99)	6.36% (4.98, 7.95)	3.07% (2.17, 4.45)	9.28% (5.72, 14.24)	14.13% (11.99, 16.76)	15.87% (13.17, 19.14)
		Falling/diving		4.22% (1.58, 9.86)	5.42% (2.61, 9.79)		5.69% (1.77, 14.95)	2.26% (0.48, 7.36)		2.01% (0.31, 8.27)	8.11% (3.47, 15.7)
	≥ 25 g	Upright	0.51% (0.28, 0.87)	0.51% (0.31, 0.82)	0.62% (0.39, 1.03)	0.41% (0.20, 0.81)	0.09% (0.04, 0.20)	0.03% (0.01, 0.08)	0.59% (0.31, 1.12)	0.92% (0.55, 1.54)	1.20% (0.77, 1.92)
		Bent at waist	0.56% (0.31, 1.01)	0.64% (0.43, 0.97)	0.66% (0.43, 1.02)	0.59% (0.29, 1.19)	0.27% (0.17, 0.45)	0.09% (0.05, 0.18)	0.50% (0.22, 1.06)	1.00% (0.69, 1.49)	1.21% (0.81, 1.79)
		Falling/diving		0.16% (0.03, 0.69)	0.25% (0.07, 0.70)		0.23% (0.04, 1.16)	0.06% (0.01, 0.35)		0.05% (0.00, 0.42)	0.39% (0.10, 1.2)
	≥ 55 g	Upright	0.01% (0.00, 0.04)	0.01% (0.00, 0.04)	0.02% (0.01, 0.05)	0.01% (0.00, 0.03)	0.00% (0.00, 0.00)	0.00% (0.00, 0.00)	0.01% (0.00, 0.05)	0.02% (0.01, 0.08)	0.03% (0.01, 0.10)
		Bent at waist	0.01% (0.00, 0.04)	0.02% (0.01, 0.04)	0.02% (0.01, 0.05)	0.01% (0.00, 0.05)	0.00% (0.00, 0.01)	0.00% (0.00, 0.00)	0.01% (0.00, 0.05)	0.03% (0.01, 0.08)	0.04% (0.01, 0.10)
		Falling/diving		0.00% (0.00, 0.03)	0.01% (0.00, 0.03)		0.00% (0.00, 0.04)	0.00% (0.00, 0.01)		0.00% (0.00, 0.01)	0.01% (0.00, 0.04)

Falling/diving–upright was removed from the women’s models owing to limited observations resulting in Hessian matrix singularity; therefore, no results were reported. Median exceedance probabilities and 95% confidence intervals are reported

*Rec.* recorded

**Table 5:** Peak angular acceleration exceedance probabilities (recorded, ≥ 2000 rad/s^2^ and ≥ 4000 rad/s^2^) for women’s rugby union players by ball-carrier–tackler body position interaction for all tackle, ball-carrier and tackler head-acceleration events

			Tackler body position								
			Mean probability			Ball-carrier probability			Tackler probability		
			Upright	Bent at waist	Falling/diving	Upright	Bent at waist	Falling/diving	Upright	Bent at waist	Falling/diving
Ball-carrier body position	Rec.	Upright	10.04% (7.49, 12.96)	8.52% (6.54, 10.87)	8.87% (7.07, 10.99)	9.00% (5.88, 13.12)	3.11% (1.87, 5.16)	1.60% (0.88, 2.86)	11.07% (7.85, 15.18)	13.93% (10.71, 18.06)	16.28% (12.71, 20.10)
		Bent at waist	10.05% (7.29, 13.60)	10.51% (8.92, 12.27)	9.41% (7.67, 11.38)	10.13% (6.57, 15.30)	6.52% (5.14, 8.16)	3.22% (2.28, 4.57)	10.19% (6.26, 15.27)	14.52% (12.39, 16.97)	15.7% (12.97, 18.69)
		Falling/diving		3.87% (1.30, 9.31)	5.40% (2.94, 9.77)		5.27% (1.49, 14.36)	2.33% (0.52, 7.74)		2.15% (0.27, 8.69)	8.05% (3.83, 15.94)
	≥ 25 g	Upright	0.62% (0.35, 1.00)	0.57% (0.34, 0.91)	0.69% (0.41, 1.04)	0.51% (0.25, 1.00)	0.10% (0.04, 0.23)	0.04% (0.01, 0.09)	0.71% (0.38, 1.28)	1.04% (0.63, 1.68)	1.33% (0.81, 2.07)
		Bent at waist	0.62% (0.35, 1.08)	0.71% (0.47, 1.02)	0.68% (0.45, 1.03)	0.61% (0.30, 1.25)	0.30% (0.19, 0.48)	0.10% (0.05, 0.19)	0.62% (0.28, 1.28)	1.11% (0.76, 1.59)	1.25% (0.84, 1.85)
		Falling/diving		0.15% (0.03, 0.65)	0.27% (0.09, 0.75)		0.22% (0.03, 1.11)	0.06% (0.01, 0.42)		0.06% (0.00, 0.50)	0.43% (0.12, 1.34)
	≥ 55 g	Upright	0.03% (0.01, 0.08)	0.03% (0.01, 0.08)	0.05% (0.02, 0.11)	0.03% (0.01, 0.08)	0.00% (0.00, 0.01)	0.00% (0.00, 0.00)	0.04% (0.01, 0.11)	0.07% (0.03, 0.16)	0.09% (0.04, 0.21)
		Bent at waist	0.03% (0.01, 0.09)	0.04% (0.02, 0.09)	0.04% (0.02, 0.10)	0.03% (0.01, 0.10)	0.01% (0.01, 0.03)	0.00% (0.00, 0.01)	0.03% (0.01, 0.11)	0.07% (0.03, 0.16)	0.09% (0.04, 0.19)
		Falling/diving		0.01% (0.00, 0.04)	0.01% (0.00, 0.06)		0.01% (0.00, 0.09)	0.00% (0.00, 0.02)		0.00% (0.00, 0.03)	0.02% (0.00, 0.11)

Falling/diving–upright was removed from the women’s models owing to limited observations resulting in Hessian matrix singularity; therefore, no results were reported. Median exceedance probabilities and 95% confidence intervals are reported

*Rec.* recorded

#### Mean Player Probability as a Function of Ball-Carrier and Tackler Body Position

The highest mean exceedance probabilities for men’s tackle events were observed when the ball-carrier was falling/diving and the tackler was upright, though this was not different to any other body position interactions. When both players were bent at the waist, probabilities of recorded HAEs for PLA and PAA were higher than from BC upright–T upright tackles and BC upright–T falling/diving, BC bent-at-waist–T falling/diving, BC falling-diving–T bent-at-waist and BC falling/diving–T falling/diving (Tables 2, 3). Higher tackle HAE probabilities were also observed for BC bent-at-waist–T upright and BC upright–T bent-at-waist compared with BC upright–T falling/diving.

No differences for higher magnitude (PLA > 55 g) HAE probability were identified between body position interactions in men. Similarly, no differences were observed for tackle probabilities in women’s competitions.

#### Probability of a Ball-Carrier HAE as a Function of Ball-Carrier and Tackler Body Position

For men’s ball-carriers, the exceedance probability was highest when the tackler was upright and the ball-carrier was either bent at the waist or falling/diving. HAE probabilities with BC falling/diving–T upright were higher than all other body position interactions except for BC bent-at-waist–T upright. BC bent-at-waist–T upright resulted in greater HAE probabilities than BC upright–T bent-at-waist, BC upright–T falling/diving and BC bent-at-waist–T falling/diving (Tables 2, 3). These differences were consistent across all PLA and PAA thresholds for men.

HAE exceedance probabilities for the ball-carrier in women’s competitions tended to be greater when the tackler was upright. BC upright–T upright and BC bent-at-waist–T upright tackles were higher compared with BC upright–T bent-at-waist, BC upright–T falling/diving and BC bent-at-waist–T falling/diving. These differences were consistent for recorded, ≥ 25 g and ≥ 2000 rad/s^2^ (Tables 4, 5). No differences were identified ≥ 55 g (Table 4). BC upright–T falling/diving was lower than BC upright–T upright, BC bent-at-waist–T upright and BC bent-at–waist-T bent-at-waist for ≥ 4000 rad/s^2^ (Table 5).

#### Probability of a Tackler HAE as a Function of Ball-Carrier and Tackler Body Position

For men, the highest exceedance probabilities for the tackler were observed when bent at the waist against a ball-carrier who was either upright or bent at the waist. These were meaningfully different compared with when the ball-carrier was in the same position, either upright or bent at the waist, and the tackler was upright. Differences were observed for recorded, ≥ 25 g and ≥ 2000 rad/s^2^ (Tables 2, 3). Meaningful differences between BC upright–T bent-at-waist and BC bent-at-waist–T bent at waist, and BC falling/diving–T bent-at-waist were observed at all PAA and PLA thresholds for men.

For women’s tacklers, the greatest exceedance probabilities were observed when the tackler was falling/diving and the ball-carrier was either upright or bent at the waist (Tables 4, 5). These were not different to when the ball-carrier was in a similar position and the tackler was either upright or bent at the waist. Lower probabilities were observed for falling/diving–bent-at-waist compared with upright–bent-at-waist and falling/diving and bent-at-waist–bent-at-waist and falling/diving for all PLA and PAA thresholds.

## Discussion

The aim of this study was to describe the mean player and role-specific probability of men’s and women’s rugby union players experiencing an HAE during the tackle event by differing tackle heights and body positions. These findings have important implications for HAE risk reduction in the game, since they agree with previous research on head injury and concussion risk in some respects, whilst differing in others. Consideration of the tackle characteristics associated with the increased chance of a ball-carrier and tackler receiving an HAE from the initial collision and the specific differences between roles, in addition to the current body of knowledge on concussion and head injury assessments (HIAs), are therefore important for future injury prevention strategies.

### Tackle Height

The findings of the present study are broadly in line with previous research suggesting high tackles result in greater odds of concussion [23]. Whilst tackles to the head/neck are relatively infrequent across the men’s and women’s game (~ 2% of tackle events), the recent focus on increased sanctions of high tackles and the adoption of rule changes in rugby union could support the reduction of initial HAEs by reducing impact to the head/neck of the ball-carrier [21, 39].

With respects to HAEs, rather than head injuries, our findings suggest that the current prevention strategies to lower the maximum legal tackle height are more likely to benefit the ball-carrier, where lower tackles reduce their chance of receiving an initial HAE. Although the probability of an HAE being observed in this study was lower than those previously reported in similar cohorts [27], our findings confirm the suggestion initial impact to the upper body and upper torso results in greater head kinematics of the ball-carrier compared with the lower body and lower torso, respectively [40, 41]. It should be of note that the ball-carrier is still subject to HAE when direct contact is not made to the head/neck, especially at the shoulder, most likely because of the sudden change in momentum during the tackle. Training interventions such as neck strength could provide some benefit to reducing indirect HAEs [42], although future research is required to assess the relationship between changes in neck strength and HAEs in the tackle, as current evidence is conflicting.

In contrast to the ball-carrier, the HAE probabilities for tacklers were greater for upper leg and lower leg contacts compared with torso and upper body contacts. Head-to-hip and head-to-knee events are considered a potential risk to the tackler for HIA and concussion, although they were found to have lower odds and rates of occurrence compared with head-head impacts [24, 43]. Our finding that tacklers have a greater probability of HAEs when contacting the ball-carrier at the upper leg compared with the shoulder may be an important consideration given the increased number of tackler concussions in law trials evaluating a reduction in the legal tackle height [25]. Therefore, whilst initiatives are currently in place to lower the initial point of impact away from the head of the ball-carrier, the probability of a tackler receiving an HAE may not be reduced; consequently, the total number of HAEs observed across the game could remain similar. Despite the lack of change in the probability of a tackler receiving an HAE, the primary outcome of any intervention aiming to reduce head injury should therefore be to avoid initial impact to the ball-carriers head/neck to minimise the number of HAEs. Future research may also wish to consider how such changes may impact other injuries, for example, if lowering the tackle height increases the number of lower limb injuries to the ball-carrier. Taking a systems-based approach to all types of severe injuries that occur in the tackle and their causal mechanisms should be considered in the future so that there are no unintended consequences as a result of changes to the tackle event.

### Body Position

An upright tackler is currently considered the highest risk of head injuries in men’s rugby union, with 1.4 times greater incidence of an HIA in men’s rugby union compared with bent-at-the-waist tacklers [24]. This is proposed to be due to the higher likelihood of a head-to-head or head-to-shoulder contact which puts both the ball-carrier and tackler at risk of sustaining a head injury [23, 24, 43]. The finding that HAE probability was greatest from two bent players differs from previous research showing that head injury risk is greatest when tacklers are upright. This could be due to the increased likelihood of both the ball-carrier and tacklers head making impact to any part of the opposition players body, whereas a more upright ball-carrier and tackler could leave more space for both heads to be positioned around or over the shoulder/torso of the opposition player. For instance, if both players are bent-at-the-waist, as may occur when a ball-carrier is dipping into the tackle, it is possible that head-to-head/shoulder could still occur in addition to head-to-torso/upper leg, increasing the overall likelihood of an HAE.

Similarly, a women’s ball-carrier was observed to have higher risk of an HAE when the tackler was upright. However, the tackler had similar probabilities of an HAE unless the ball-carrier was falling/diving; in which case, the probabilities were lower. Once again, these are likely situations where players may or may not find their head in space to avoid impact with the opposition player’s body. Given the current initiatives in place to encourage the tackler to bend at the waist to prevent head injuries [21, 25], and our finding that the greatest HAE probability occurred at the most frequently occurring body position interaction (i.e. bent-at-the-waist, men, 55.30% and women, 46.20%), an upright body position may be favoured to reduce HAEs, whilst being bent at the waist is preferable for reducing head injuries. Further investigation is warranted into how HAEs occur in these positions to reduce the accumulation of HAEs from the tackle through either tackle technique interventions or law changes. Consideration of the differences between sexes may also be required given our finding that no difference in HAE risk was observed between any body positions in women, as interventions may not be reflected equally.

It should be noted that when considering higher magnitude HAEs (≥ 55 g and ≥ 4000 rads/s^2^), differences for all ball-carrier and tackler body positions were not clear. Although recent research has identified relationships between HAE magnitude and HIAs [44], it is possible that there are differences in characteristics of tackles that lead to clinical outcomes and lower magnitude HAEs. It is suggested that governing bodies take note of both the accumulation of non-concussive HAEs (i.e. not identified as a possible acute head injury) and the clinical outcomes, given the potential association between the accumulation of HAEs and negative long-term brain health outcomes.

## Limitations

Whilst this study is the first to look at head contacts and tackle mechanisms in both elite men’s and women’s rugby union players, it is not without limitations. First, as a consequence of the convenience sample, the sample is non-random, and although it includes players from a number of leagues, it may not be representative of the playing population and could be subject to selection bias. Second, because of infrequent tackle mechanisms in the sample gathered, there were a low number of observations for certain categories (e.g. BC falling/diving–T upright tackles in the women’s competitions; *n* = 5), which had to be removed. In other cases, confidence interval could be inflated as a consequence of sparse data bias, e.g. BC upright–T falling/diving for men [45]. Similarly, owing to the small number of events per variable, it was not possible to explore the multiple relationships between body positions, tackle heights and HAEs via interaction terms. The initial point of impact when both players are bent-at-the-waist, rather than just the body positions on their own, could be an important consideration explaining the findings, for example. Furthermore, the tackle is a dynamic skill and other mechanisms and contextual factors that form a causal path within the data (e.g. tackler body position → tackle height → HAE). Therefore, to fully understand the mechanistic role of multiple tackle characteristics on HAEs, especially secondary and grounding HAEs, rather than univariable approaches, multivariable analysis methods may be appropriate when larger datasets are available to consider the counterfactual distribution of probabilities to support policy change. Finally, it is possible that exceedance probabilities lower than 25 g are underestimated owing to the potential linear trigger bias imposed by a trigger threshold of 8 g [46, 47].

## Conclusions

The current study identified the relationships between tackle height and body positions and HAEs in the rugby union tackle. In agreement with the previous tackle height and head injury literature, the greatest probability of HAEs during the tackle is when initial contact is made to the ball-carrier’s head/neck (higher contact). However, role-specific differences highlight that whilst lower tackle heights have a lower probability of the ball-carrier receiving an HAE, the tackler has a similar probability compared with a higher tackle height. In addition, it is possible that when players are bent at the waist, there is a greater likelihood of receiving an HAE when compared with upright–upright tackles. Therefore, tackles where the players are upright, whilst avoiding initial impact to the ball-carriers head/neck, may pose the least risk for players receiving an HAE. To date, the current player welfare initiatives have been based on HIA and concussion data; however, given the potential importance of accumulation of HAE in long term brain health, future welfare strategies should also consider HAE mechanisms as part of those that result in clinical outcomes.

## Policy Implications

Consideration of policies and coaching interventions with regard to tackle height and player body position should be contemplated. Focus should be on reducing initial impact to the ball-carrier’s head/neck, whilst coaching interventions specific to the tackler may want to focus on improving technique around the lower leg or targeting the shoulder and torso to reduce HAEs. Policies or techniques should also focus on players maintaining a more upright posture where possible whilst avoiding contact to the ball-carrier’s head to reduce HAEs.

## Supplementary Information

Below is the link to the electronic supplementary material.

Supplementary file1 (PDF 654 KB)

## Declarations

### Availability of data and material

All data relevant to the study are included in the article or uploaded as supplementary information. Anonymised data are available upon reasonable request.

### Code availability

Code for analyses will be available upon reasonable request.
