# Supplementary material for: Evaluating the Probability of Head Acceleration Events in Elite Men’s and Women’s Rugby Union Match-Play: The Impact of Tackle Height and Body Position
Source: Sports Med. 2025 May 7;55(10):2641–57. doi: 10.1007/s40279-025-02241-2 (PMC12513871; doi:10.1007/s40279-025-02241-2)
Supplement: Supplementary file 1 — Supplementary file1 (PDF 654 KB) [file 40279_2025_2241_MOESM1_ESM.pdf]

Journal: Sports Medicine

Title: Evaluating the Probability of Head Acceleration Events in Elite Men's and Women's Rugby Union Match-Play: The Impact of Tackle Height and Body Position

Cameron Owen[1,2],* Greg Roe[1], James Tooby[1], Thomas Sawczuk[1,3], James Brown[1,4,5], Matt Cross[1,6], Éanna Falvery[7,8], Sharief Hendricks[1,4], Simon Kemp[9,10], Lindsay Starling[7,11,12], Keith Stokes[9,11,12], Ross Tucker[5,7], Ben Jones[1,2,4,6,13]

[1] Carnegie Applied Rugby Research (CARR) Centre, Carnegie School of Sport, Leeds Beckett University, Leeds, UK.

[2] England Performance Unit, Rugby Football League, Manchester, UK.

[3] Obesity Institute, Leeds Beckett University, Leeds, UK.

[4] Division of Physiological Sciences, Department of Human Biology, Faculty of Health Sciences, University of Cape Town, Cape Town, South Africa.

[5] The Division of Sport and Exercise Medicine (DiSEM), Department of Exercise, Sport and Lifestyle Medicine, Faculty of Medicine and Health Sciences, Stellenbosch University

[6] Premiership Rugby, London, UK.

[7] World Rugby, Dublin, Ireland.

[8] School of Medicine & Health, University College Cork, Cork, Ireland.

[9] Rugby Football Union, Twickenham, UK.

[10] London School of Hygiene and Tropical Medicine, London, United Kingdom

[11] Centre for Health and Injury and Illness Prevention in Sport, University of Bath, Bath, UK.

[12] UK Collaborating Centre on Injury and Illness Prevention in Sport (UKCCIIS), University of Bath, Bath, UK.

[13] Faculty of Health Sciences, School of Behavioural and Health Sciences, Australian Catholic University, Brisbane, Queensland, Australia.

* Corresponding author email: Cameron.owen@leedsbeckett.ac.uk

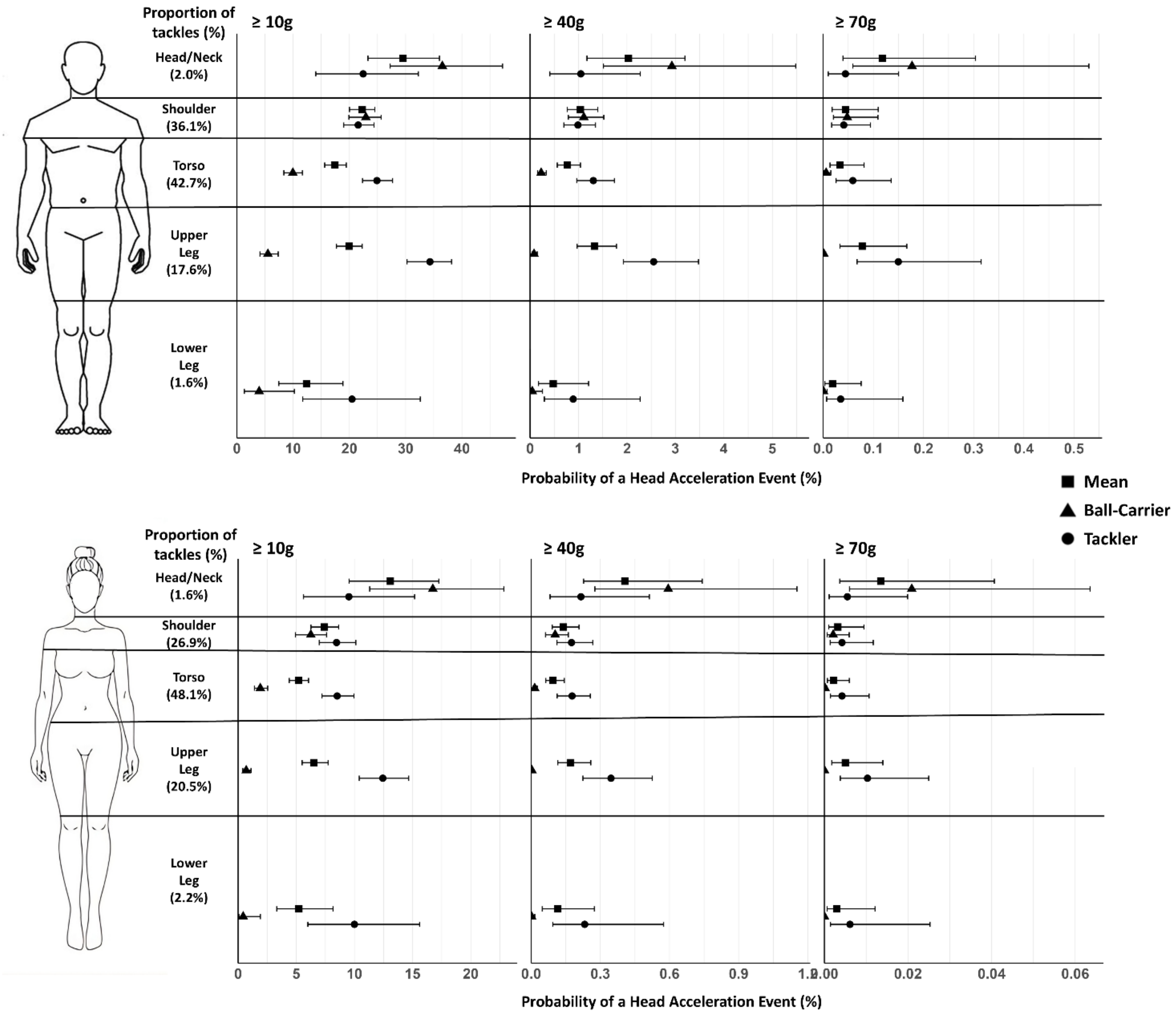

Supplementary 1. Peak linear acceleration exceedance probabilities for men's (≥10g, ≥40g and ≥70g) and women's (≥10g, ≥40g and ≥70g) rugby union players by tackle height for all tackle, ball-carrier and tackler head acceleration events. Shape position indicates the median exceedance probability, with 95% confidence intervals show by the error bars. Note all x-axis are different.

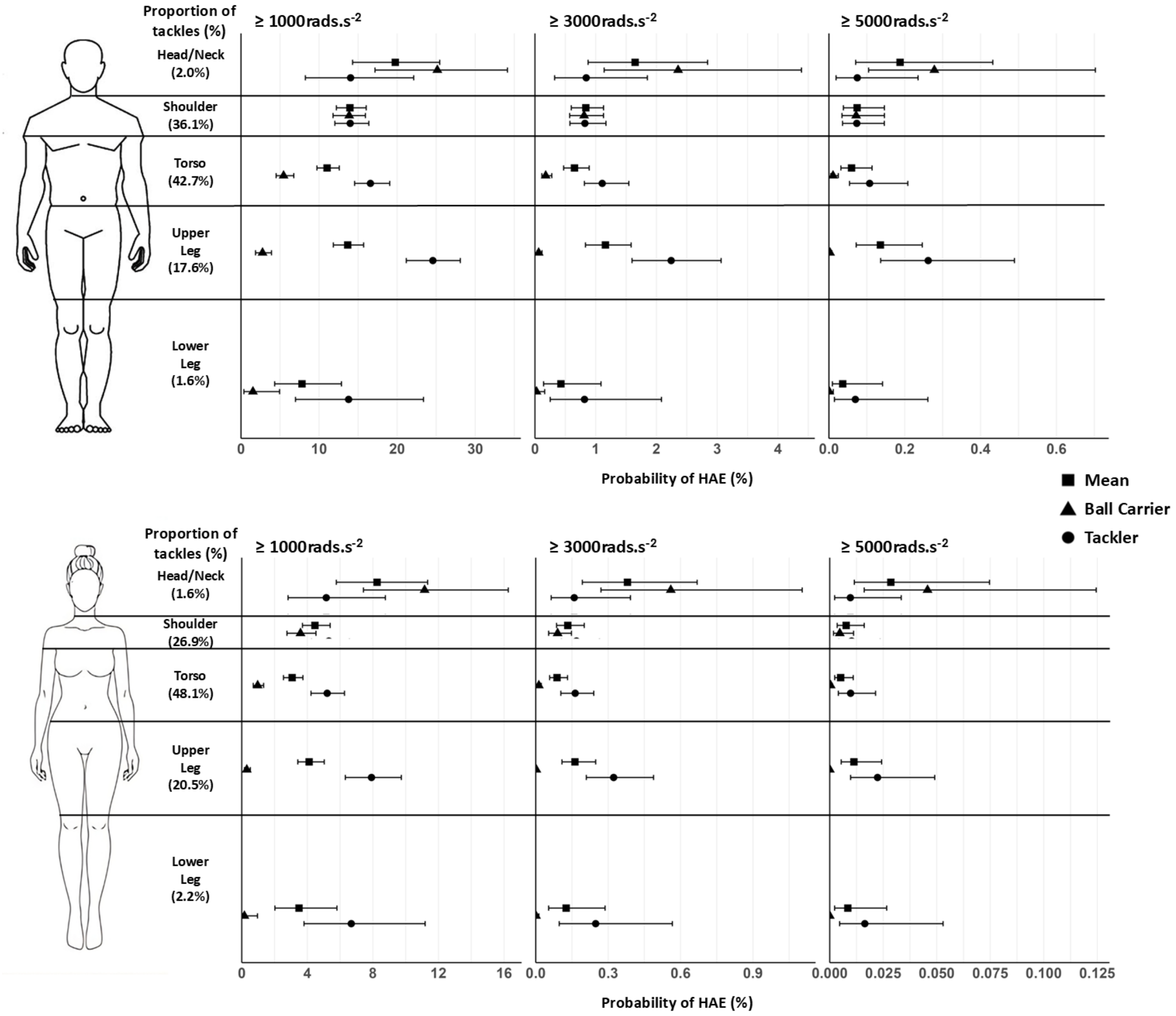

Supplementary 2. Peak angular acceleration exceedance probabilities for men's (≥21,000rads.s^{-2}, ≥3,000rads.s^{-2} and ≥5,000rads.s^{-2}) and women's (≥1,000rads.s^{-2}, ≥3,000rads.s^{-2} and ≥5,000rads.s^{-2}) rugby union players by tackle height for all tackle, ball-carrier and tackler head acceleration events. Shape position indicates the median exceedance probability, with 95% confidence intervals show by the error bars. Note all x-axis are different.

Supplementary 3. Peak linear acceleration exceedance probabilities (≥10g, ≥40g and ≥70g) for men's rugby union players by ball-carrier – tackler body position interaction for all tackle, ball-carrier and tackler head acceleration events. Large font 'Tackler' indicates the body position of the tackler, whilst the small font 'Tackler' indicates the probability of a head acceleration event occurring to the tackler for a given body position interaction. Median exceedance probabilities and 95% confidence intervals are reported.

		Tackler Body Position								
		Mean Probability			Ball-Carrier Probability			Tackler Probability		
Ball-Carrier Body Position		Upright	Bent at Waist	Falling/Diving	Upright	Bent at Waist	Falling/Diving	Upright	Bent at Waist	Falling/Diving
≥10g	Upright	16.45% (13.56, 19.59)	19.77% (17.02, 22.73)	12.77% (10.01, 15.95)	14.61% (11.35, 18.62)	10.18% (7.77, 13.14)	3.78% (2.05, 6.38)	18.29% (14.42, 23.11)	29.52% (25.01, 34.38)	21.76% (16.75, 27.72)
≥10g	Bent at Waist	19.54% (16.33, 23.07)	22.46% (20.74, 24.15)	17.14% (14.25, 20.04)	20.44% (15.94, 25.92)	16.84% (15.11, 18.82)	6.93% (4.65, 10.08)	18.79% (14.54, 23.6)	28.22% (25.82, 30.57)	27.37% (22.63, 32.97)
≥10g	Falling/Diving	30.75% (17.97, 43.78)	13.48% (9.77, 17.90)	12.86% (7.93, 19.15)	43.51% (22.11, 65.31)	13.79% (8.80, 20.34)	8.76% (3.88, 17.24)	17.56% (6.43, 36.38)	12.96% (8.54, 19.34)	16.75% (9.50, 26.94)
≥40g	Upright	0.70% (0.45, 1.03)	1.19% (0.84, 1.68)	0.61% (0.37, 0.97)	0.55% (0.33, 0.88)	0.29% (0.17, 0.46)	0.05% (0.02, 0.14)	0.83% (0.52, 1.35)	2.13% (1.46, 2.99)	1.16% (0.67, 1.91)
≥40g	Bent at Waist	0.95% (0.64, 1.37)	1.32% (1.03, 1.64)	0.98% (0.65, 1.42)	1.03% (0.62, 1.67)	0.72% (0.53, 0.95)	0.15% (0.07, 0.31)	0.88% (0.51, 1.44)	1.95% (1.52, 2.45)	1.84% (1.17, 2.69)
≥40g	Falling/Diving	2.94% (0.90, 7.26)	0.49% (0.27, 0.83)	0.48% (0.20, 1.13)	4.91% (1.21, 13.71)	0.50% (0.22, 1.06)	0.23% (0.05, 0.74)	0.77% (0.13, 3.38)	0.44% (0.20, 0.96)	0.70% (0.24, 1.83)
≥70g	Upright	0.03% (0.01, 0.08)	0.08% (0.03, 0.17)	0.03% (0.01, 0.09)	0.02% (0.01, 0.07)	0.01% (0.00, 0.03)	0.00% (0.00, 0.01)	0.04% (0.01, 0.11)	0.15% (0.06, 0.32)	0.07% (0.02, 0.16)
≥70g	Bent at Waist	0.05% (0.02, 0.12)	0.08% (0.04, 0.17)	0.06% (0.02, 0.14)	0.06% (0.02, 0.15)	0.03% (0.01, 0.08)	0.00% (0.00, 0.02)	0.04% (0.02, 0.12)	0.13% (0.06, 0.27)	0.12% (0.05, 0.28)
≥70g	Falling/Diving	0.26% (0.04, 1.15)	0.02% (0.01, 0.06)	0.02% (0.00, 0.08)	0.46% (0.07, 2.25)	0.02% (0.00, 0.07)	0.01% (0.00, 0.04)	0.04% (0.00, 0.33)	0.02% (0.01, 0.06)	0.03% (0.01, 0.14)

Supplementary 4. Peak angular acceleration exceedance probabilities (≥1,000rads.s^{-2}, ≥3,000rads.s^{-2}and ≥5,000rads.s^{-2}) for men's rugby union players by ball-carrier – tackler body position interaction for all tackle, ball-carrier and tackler head acceleration events. Large font 'Tackler' indicates the body position of the tackler, whilst the small font 'Tackler' indicates the probability of a head acceleration event occurring to the tackler for a given body position interaction. Median exceedance probabilities and 95% confidence intervals are reported.

		Tackler Body Position								
		Mean Probability			Ball-Carrier Probability			Tackler Probability		
Ball-Carrier Body Position		Upright	Bent at Waist	Falling/Diving	Upright	Bent at Waist	Falling/Diving	Upright	Bent at Waist	Falling/Diving
≥1,000 rads.s^{-2}	Upright	9.71% (7.70, 11.92)	13.01% (10.98, 15.16)	7.42% (5.33, 9.80)	8.19% (6.05, 11.08)	5.73% (4.23, 7.63)	1.5% (0.67, 3.06)	11.24% (8.49, 14.46)	20.13% (16.60, 24.01)	13.31% (9.75, 17.91)
	Bent at Waist	11.62% (9.38, 14.42)	13.71% (12.34, 15.25)	9.91% (7.90, 12.36)	11.54% (8.54, 15.76)	9.04% (7.69, 10.43)	3.31% (2.09, 5.08)	11.63% (8.73, 15.66)	18.42% (16.53, 20.35)	16.54% (13.00, 20.83)
	Falling/Diving	17.22% (7.85, 28.92)	7.66% (5.06, 10.87)	7.03% (3.96, 11.26)	24.46% (10.29, 44.83)	7.76% (4.62, 12.31)	4.37% (1.69, 9.87)	8.91% (2.50, 21.12)	7.24% (4.14, 11.56)	9.32% (4.62, 17.54)
≥3,000 rads.s^{-2}	Upright	0.51% (0.32, 0.81)	0.98% (0.65, 1.45)	0.44% (0.23, 0.76)	0.38% (0.22, 0.69)	0.22% (0.12, 0.38)	0.03% (0.01, 0.09)	0.65% (0.37, 1.08)	1.74% (1.16, 2.56)	0.85% (0.47, 1.47)
	Bent at Waist	0.68% (0.42, 1.07)	0.97% (0.7, 1.31)	0.66% (0.41, 1.07)	0.68% (0.38, 1.21)	0.45% (0.30, 0.66)	0.09% (0.04, 0.19)	0.68% (0.4, 1.2)	1.47% (1.10, 2.03)	1.23% (0.74, 1.96)
	Falling/Diving	1.51% (0.39, 4.41)	0.35% (0.17, 0.68)	0.33% (0.13, 0.75)	2.46% (0.56, 8.38)	0.35% (0.15, 0.78)	0.15% (0.03, 0.52)	0.43% (0.06, 1.90)	0.32% (0.12, 0.71)	0.48% (0.14, 1.44)
≥5,000 rads.s^{-2}	Upright	0.03% (0.01, 0.09)	0.09% (0.04, 0.20)	0.03% (0.01, 0.09)	0.02% (0.01, 0.07)	0.01% (0.00, 0.03)	0.00% (0.00, 0.01)	0.05% (0.02, 0.11)	0.17% (0.07, 0.38)	0.07% (0.02, 0.17)
	Bent at Waist	0.05% (0.02, 0.12)	0.08% (0.04, 0.18)	0.06% (0.02, 0.14)	0.05% (0.02, 0.14)	0.03% (0.01, 0.07)	0.00% (0.00, 0.01)	0.05% (0.02, 0.14)	0.14% (0.06, 0.30)	0.11% (0.04, 0.26)
	Falling/Diving	0.16% (0.02, 0.71)	0.02% (0.01, 0.07)	0.02% (0.00, 0.07)	0.26% (0.03, 1.43)	0.02% (0.01, 0.08)	0.01% (0.00, 0.05)	0.03% (0.00, 0.21)	0.02% (0.00, 0.07)	0.03% (0.01, 0.14)

Supplementary 5. Peak linear acceleration exceedance probabilities (≥10g, ≥40g and ≥70g) for men's rugby union players by ball-carrier – tackler body position interaction for all tackle, ball-carrier and tackler head acceleration events. Falling/diving-upright was removed from the women's models due to limited observations resulting in Hessian matrix singularity, so no results were reported. Large font 'Tackler' indicates the body position of the tackler, whilst the small font 'Tackler' indicates the probability of a head acceleration event occurring to the tackler for a given body position interaction. Median exceedance probabilities and 95% confidence intervals are reported.

		Tackler Body Position								
		Mean Probability			Ball-Carrier Probability			Tackler Probability		
Ball-Carrier Body Position		Upright	Bent at Waist	Falling/Diving	Upright	Bent at Waist	Falling/Diving	Upright	Bent at Waist	Falling/Diving
≥10g	Upright	5.18% (3.61, 7.23)	4.63% (3.35, 6.28)	5.03% (3.87, 6.70)	4.38% (2.75, 7.00)	1.43% (0.78, 2.47)	0.65% (0.30, 1.25)	5.77% (3.78, 8.56)	7.84% (5.68, 10.69)	9.45% (7.23, 12.4)
	Bent at Waist	5.49% (3.77, 7.74)	5.82% (4.72, 7.14)	5.53% (4.34, 7.04)	5.81% (3.61, 8.98)	3.31% (2.48, 4.39)	1.46% (0.98, 2.24)	5.11% (2.88, 8.51)	8.30% (6.79, 10.16)	9.48% (7.48, 11.86)
	Falling/Diving		2.13% (0.70, 5.84)	2.86% (1.25, 5.69)		2.91% (0.77, 8.75)	1.03% (0.19, 3.90)		0.92% (0.12, 4.5)	4.33% (1.69, 9.61)
≥40g	Upright	0.06% (0.03, 0.14)	0.07% (0.03, 0.14)	0.09% (0.04, 0.19)	0.05% (0.02, 0.13)	0.01% (0.00, 0.02)	0.00% (0.00, 0.01)	0.07% (0.03, 0.19)	0.13% (0.06, 0.27)	0.18% (0.09, 0.36)
	Bent at Waist	0.07% (0.03, 0.17)	0.09% (0.04, 0.16)	0.10% (0.05, 0.18)	0.08% (0.03, 0.20)	0.03% (0.01, 0.06)	0.01% (0.00, 0.02)	0.06% (0.02, 0.17)	0.14% (0.08, 0.26)	0.18% (0.09, 0.34)
	Falling/Diving		0.02% (0.00, 0.10)	0.03% (0.01, 0.11)		0.02% (0.00, 0.19)	0.00% (0.00, 0.04)		0.00% (0.00, 0.06)	0.05% (0.01, 0.18)
≥70g	Upright	0.00% (0.00, 0.00)	0.00% (0.00, 0.00)	0.00% (0.00, 0.00)	0.00% (0.00, 0.00)	0.00% (0.00, 0.00)	0.00% (0.00, 0.00)	0.00% (0.00, 0.00)	0.00% (0.00, 0.00)	0.00% (0.00, 0.00)
	Bent at Waist	0.00% (0.00, 0.00)	0.00% (0.00, 0.00)	0.00% (0.00, 0.00)	0.00% (0.00, 0.00)	0.00% (0.00, 0.00)	0.00% (0.00, 0.00)	0.00% (0.00, 0.00)	0.00% (0.00, 0.00)	0.00% (0.00, 0.00)
	Falling/Diving		0.00% (0.00, 0.00)	0.00% (0.00, 0.00)		0.00% (0.00, 0.00)	0.00% (0.00, 0.00)		0.00% (0.00, 0.00)	0.00% (0.00, 0.00)

Supplementary 6. Peak angular acceleration exceedance probabilities (≥1,000rads.s^{-2}, ≥3,000rads.s^{-2}and ≥5,000rads.s^{-2}) for men's rugby union players by ball-carrier – tackler body position interaction for all tackle, ball-carrier and tackler head acceleration events. Falling/diving-upright was removed from the women's models due to limited observations resulting in Hessian matrix singularity, so no results were reported. Large font 'Tackler' indicates the body position of the tackler, whilst the small font 'Tackler' indicates the probability of a head acceleration event occurring to the tackler for a given body position interaction. Median exceedance probabilities and 95% confidence intervals are reported.

Ball-Carrier Body Position		Tackler Body Position								
		Mean Probability			Ball-Carrier Probability			Tackler Probability		
		Upright	Bent at Waist	Falling/Diving	Upright	Bent at Waist	Falling/Diving	Upright	Bent at Waist	Falling/Diving
≥1,000 rads.s^{-2}	Upright	3.74% (2.56, 5.22)	3.24% (2.32, 4.53)	3.59% (2.57, 4.77)	3.27% (1.90, 5.30)	0.89% (0.47, 1.67)	0.40% (0.20, 0.82)	4.23% (2.68, 6.44)	5.62% (4.00, 8.01)	6.83% (4.97, 9.05)
	Bent at Waist	3.74% (2.49, 5.61)	4.05% (3.16, 5.07)	3.7% (2.81, 4.85)	3.77% (2.20, 6.29)	2.17% (1.61, 2.98)	0.93% (0.59, 1.47)	3.77% (2.10, 6.43)	5.9% (4.75, 7.37)	6.53% (5.00, 8.37)
	Falling/Diving		1.19% (0.32, 3.6)	1.85% (0.84, 3.90)		1.68% (0.37, 5.91)	0.62% (0.11, 2.72)		0.57% (0.05, 3.15)	2.85% (1.12, 6.48)
≥3,000 rads.s^{-2}	Upright	0.10% (0.04, 0.20)	0.10% (0.05, 0.19)	0.13% (0.06, 0.24)	0.08% (0.03, 0.19)	0.01% (0.00, 0.03)	0.00% (0.00, 0.01)	0.12% (0.05, 0.26)	0.19% (0.09, 0.37)	0.25% (0.12, 0.48)
	Bent at Waist	0.10% (0.04, 0.23)	0.12% (0.07, 0.22)	0.12% (0.06, 0.23)	0.10% (0.04, 0.25)	0.04% (0.02, 0.09)	0.01% (0.01, 0.03)	0.10% (0.03, 0.26)	0.2% (0.11, 0.36)	0.23% (0.13, 0.42)
	Falling/Diving		0.02% (0.00, 0.12)	0.04% (0.01, 0.15)		0.03% (0.00, 0.23)	0.01% (0.00, 0.07)		0.01% (0.00, 0.08)	0.06% (0.01, 0.28)
≥5,000 rads.s^{-2}	Upright	0.01% (0.00, 0.03)	0.01% (0.00, 0.03)	0.01% (0.00, 0.04)	0.01% (0.00, 0.03)	0.00% (0.00, 0.00)	0.00% (0.00, 0.00)	0.01% (0.00, 0.04)	0.02% (0.01, 0.07)	0.03% (0.01, 0.08)
	Bent at Waist	0.01% (0.00, 0.04)	0.01% (0.00, 0.04)	0.01% (0.00, 0.04)	0.01% (0.00, 0.04)	0% (0.00, 0.01)	0.00% (0.00, 0.00)	0.01% (0.00, 0.04)	0.02% (0.01, 0.07)	0.02% (0.01, 0.08)
	Falling/Diving		0.00% (0.00, 0.01)	0.00% (0.00, 0.02)		0.00% (0.00, 0.03)	0.00% (0.00, 0.01)		0.00% (0.00, 0.01)	0.01% (0, 0.03)
